# Intensity-Modulated and Image-Guided Radiotherapy in Patients with Locally Advanced Inoperable Pancreatic Cancer after Preradiation Chemotherapy

**DOI:** 10.1155/2014/452089

**Published:** 2014-10-20

**Authors:** M. Sinn, R. Ganeshan, R. Graf, U. Pelzer, J. M. Stieler, J. K. Striefler, M. Bahra, P. Wust, H. Riess

**Affiliations:** ^1^Department of Medical Oncology and Haematology, Charité, Universitätsmedizin Berlin, Augustenburger Platz 1, 13353 Berlin, Germany; ^2^Department of Radiooncology, Charité, Universitätsmedizin Berlin, Augustenburger Platz 1, 13353 Berlin, Germany; ^3^Department of General, Visceral and Transplantation Surgery, Charité, Universitätsmedizin Berlin, Augustenburger Platz 1, 13353 Berlin, Germany

## Abstract

*Background*. Radiotherapy (RT) in patients with pancreatic cancer is still a controversial subject and its benefit in inoperable stages of locally advanced pancreatic cancer (LAPC), even after induction chemotherapy, remains unclear. Modern radiation techniques such as image-guided radiotherapy (IGRT) and intensity-modulated radiotherapy (IMRT) may improve effectiveness and reduce radiotherapy-related toxicities. *Methods*. Patients with LAPC who underwent radiotherapy after chemotherapy between 09/2004 and 05/2013 were retrospectively analyzed with regard to preradiation chemotherapy (PRCT), modalities of radiotherapy, and toxicities. Progression-free (PFS) and overall survival (OS) were estimated by Kaplan-Meier curves. *Results*. 15 (68%) women and 7 men (median age 64 years; range 40–77) were identified. Median duration of PRCT was 11.1 months (range 4.3–33.0). Six patients (27%) underwent conventional RT and 16 patients (73%) advanced IMRT and IGRT; median dosage was 50.4 (range 9–54) Gray. No grade III or IV toxicities occurred. Median PFS (estimated from the beginning of RT) was 5.8 months, 2.6 months in the conventional RT group (conv-RT), and 7.1 months in the IMRT/IGRT group (*P* = 0.029); median OS was 11.0 months, 4.2 months (conv-RT), and 14.0 months (IMRT/IGRT); *P* = 0.141. Median RT-specific PFS for patients with prolonged PRCT > 9 months was 8.5 months compared to 5.6 months for PRCT < 9 months (*P* = 0.293). This effect was translated into a significantly better median RT-specific overall survival of patients in the PRCT > 9 months group, with 19.0 months compared to 8.5 months in the PRCT  <  9 months group (*P* = 0.049). *Conclusions*. IGRT and IMRT after PRCT are feasible and effective options for patients with LAPC after prolonged preradiation chemotherapy.

## 1. Introduction

The role of radiotherapy (RT) in the treatment of pancreatic cancer remains a controversial issue. For locally advanced and inoperable tumors (LAPC), RT was considered to be a reasonable therapeutic option. As this subgroup is relatively rare, however, representing about 30% of patients with pancreatic cancer [1], the possibilities for clinical investigations are limited, and recruitment in phase 3 trials was worse than expected [2]. Furthermore, the optimal technique for pancreatic irradiation has not yet been defined, as in previous studies inconsistent doses, schedules, and concomitant chemotherapies contributed to an inhomogeneous level of knowledge [3]. The side effects of outdated techniques (e.g., opposite fields) for upper abdominal radiotherapy have prevented its widespread use, and in many cases potential benefits of RT were restricted by the typical course of the disease, due to early occurrence of distant metastasis. In summary, no real standard for LAPC has so far been established [4].

The potential value of RT should be reconsidered in light of two recent innovations.

Firstly, intensity-modulated (IMRT) and image-guided (IGRT) radiotherapy have been significantly improved [5]. Modern technologies such as Tomotherapy or RapidArc result in dose distributions better adapted to arbitrary target volumes. In addition, image guidance such as cone-beam, megavoltage CT, or portal imaging allows a further reduction of safety margins. Both developments make it easier to spare the surrounding normal tissues in the upper abdomen such as kidneys, intestines, stomach, and liver and allow both an increase of the target volume dose and at the same time a lower dose in the neighboring organs. After all, these radiotherapeutic options are considered more effective and in addition more tolerable.

Secondly, the concept of induction chemotherapy for patients with LAPC contributed pragmatically to identify patients without rapid distant metastasis as especially eligible for local therapy in clinical routine [6], but type and duration of preradiation chemotherapy (PRCT) have not yet been defined. In fact, the small amount of data available which analyzes patients with LAPC after induction chemotherapy and subsequent RT is controversial and even less is available for RT using image-guided techniques [5] or the role of intraoperative radiotherapy [7]. Former data reveal a reduction in the total volume irradiated and suggest a potentially decreased chance of marginal miss as well as a better reproducibility of gastrointestinal filling by the use of IGRT in LAPC. IMRT seems to reduce acute and late side effects and the dose to organs at risk. IMRT or IGRT may allow for the use of hypofractionation or dose escalation or both [8].

Our single-center experience may help to clarify the feasibility and efficacy of these concepts.

## 2. Methods

We performed a retrospective analysis of consecutive patients with LAPC treated between September 2005 and May 2013 in the Oncological Department of the Charité-Universitätsmedizin Berlin, Germany. Patients were considered eligible for the evaluation if they had unresectable pancreatic cancer (primary or locally recurrent) documented by CT scan, no evidence of distant metastases (usually verified by FDG-PET), and had undergone chemotherapy followed by radiotherapy. Chemotherapy duration had been selected by the treating physician; the indication for radiotherapy was based on individual decisions after interdisciplinary discussions and those with the individual patient.

Patient's characteristics, treatment courses, and relevant toxicities were collected. Median progression-free survival (PFS) was estimated according to Kaplan and Meier. Toxicities were evaluated using the Common Terminology Criteria for Adverse Events (CTCAE), version 3.0.

RT-specific PFS was defined as duration from the start of RT until tumor progression, RT-specific overall survival (OS) was estimated from the start of RT until death. General PFS was measured from the beginning of first-line palliative chemotherapy to tumor progression; analogous general OS was estimated from the date of primary diagnosis of unresectable disease until death.

Comparisons between subgroups were performed using the log-rank test. Hazard ratios were determined using cox regression. Results were considered significant at *P* ≤ 0.05. The database was closed on May 2013.

All data analyses were performed using IBM SPSS Statistics, version 19 (German).

## 3. Results

### 3.1. Patient Characteristics

22 patients were identified, 15 women (68%) and 7 men. Median age was 64 years at the beginning of radiotherapy (range 40–77). 16 patients (73%) had tumor localization in the head of the pancreas, 5 (22%) in the body, and 1 (5%) in the tail. 10 patients (45.5%) underwent resection of the primary tumor, 8 received adjuvant therapy, and the remaining 12 patients (54.5%) had primary inoperable disease. All but three patients had an index of over 80% on the Karnofsky Performance Scale.

All patients had previously received chemotherapy. In 13 patients (59%) chemotherapy was followed immediately by radiation therapy; in 9 patients (41%) there was a delay between chemotherapy and radiation therapy with a median of 54 days (range 4–635 days). All patients received gemcitabine-based chemotherapy before radiation therapy (in 20 pts as monotherapy, in 2 pts as combination therapy), and 5 patients had undergone more than one previous chemotherapy regimen. For details of preceding chemotherapy please refer to Table 1.

### 3.2. Radiotherapy

At the beginning of our study, 6 patients underwent conventional 3D-conformal RT; later 16 (72.7%) patients (72.7%) were treated with advanced IMRT and IGRT techniques (Tomotherapy, Novalis, RapidArc). 10 patients had radiotherapy alone; the other 12 patients (54.5%) had radiotherapy combined with chemotherapy (6 received gemcitabine and 6 capecitabine); for details see Table 1.

Median duration from the beginning of PRCT and the start of RT was 11.1 months (range 4.3–33.0); in 9 (41%) patients the duration of preradiation chemotherapy was less than 9 months. As documented by preceding CT scan or PET-CT, no patients had distant metastasis prior to beginning RT.

The median applied dosage was 50.4 (range 9–54) Gray and 17 patients (77%) were able to complete the planned RT. 10 patients received an integrated boost (60.2–64.5).

The reason for premature end of RT was systemic progression in 4 patients (2 with rapid deaths). 1 patient had to stop RT due to intercurrent cholestasis. 4 of the patients (67%) undergoing conventional RT had to stop RT prematurely, in contrast to only 1 (6%) of the 16 patients receiving IGRT.

The most common toxicity during RT was fatigue (36.3%); the most important adverse advents were gastrointestinal in nature: diarrhea (27.2%), appetite loss (22.7%), nausea (18.1%), and weight loss (18.1%); no grade III or IV or relevant hematotoxicity occurred.

Local lymph nodes, if involved, were included in the target volume. IMRT and IGRT were performed either with the stereotactic linear accelerator Novalis (using ExacTrac for image guidance) for small tumors (<6 cm extension) or with the Tomotherapy system (using megavoltage CT, MV-CT for image guidance) for larger tumors. A safety margin of 5 mm from the clinical target volume (CTV) to the planning target volume (PTV) was typically defined.

**Figure 1:**
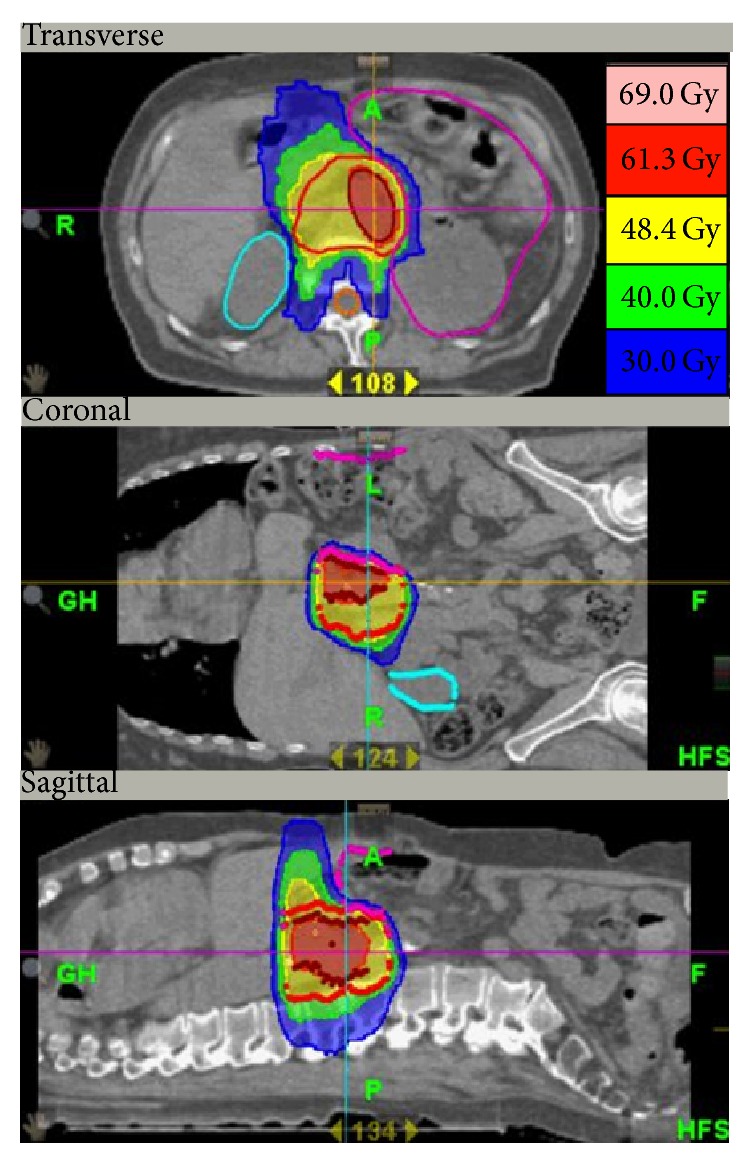
Radiation plan. Representative dose distribution of a Tomotherapy plan for a pancreatic recurrency, given in three orthogonal planes: the dark red contour delineates the macroscopic tumor (ascertained by PET) with a prescribed dose of 64.5 Gy. The planning target volume (PTV) is light red and includes the adjacent lymph nodes. The prescribed dose is 51 Gy. Dose coverage of >95% is achieved. The surrounding normal tissues liver, kidneys (light blue), intestine (magenta), and spinal cord (orange) are particularly spared. In this patient only a small part of the intestine (<100 mL) is exposed to a dose > 30 Gy (blue region). Note the high conformality as well as the steep dose gradient from 60 below 30 Gy in selected directions.

The planning CT was fused with the FDG-tracer distribution (PET) and MRI, if available. The gross tumor volume (GTV) for the boost was identified in the CT (supported by contrast media) utilizing the FDG-uptake. The CTV included a safety margin and adjacent lymph node regions. The CTV dose prescription was 51 Gy in 30 fractions to CTV and up to 64.5–66 Gy in 30 fractions to GTV as simultaneous integrated boost (SIB). Normal surrounding tissues were also specified. Dose distribution optimization was performed with restrictions for intestines/stomach (10 mL > 50 Gy, 100 mL >30 Gy), kidneys (10% > 15–18 Gy), liver (100 mL > 30 Gy), and spinal canal (<36 Gy); see Figure 1.

In the period from 2005 to 2007, maximum doses of 45–50.4 Gy (25–28 × 1.8 Gy) were permitted/tolerated in the target volumes using 3D-conformal radiotherapy. With regard to acute and late toxicity, limiting organs were intestinal structures and kidneys. Using modern radiotherapy techniques we therefore achieved radiation doses of 10–16 Gy higher in the macroscopic tumor with lower toxicity and risks.

Both the high performance of the dose distribution and image guidance are of crucial importance for the therapeutic ratio of radiotherapy. In particular, the image guidance implemented in the Tomotherapy system using MV-CT was suitable for radiotherapy of the upper abdomen. The CT-scans show not only bony structures (spine), but also organs such as kidneys or liver and large vessels such as the abdominal aorta or vena cava. Based on these landmarks, the tumor region was identified and recognized in the MV-CT. A reliable adjustment of the best patient position was achieved using this comprehensive anatomical information.

As organ motion might reduce the precision, the dominant source of misalignment is the* interfractional dislocation* or shift, which is caused by different organ fillings (stomach, intestine) and the from day to day variable equilibrium breathing position. For every fraction, the correct position of the tumor involved region was identified in the MV-CT utilizing the anatomical landmarks described above. The individual strategy was specified by the radiation oncologist considering all available clinical details. An additional but less important error source is the* intrafractional dislocation*, which is usually due to the cyclic breathing motion, but can be considerably increased by a shift of the resting respiratory position. Therefore, we instructed the patient to keep a quiet respiration without large excursions to limit this intrafractional motion, during the planning CT as well as during each radiotherapy fraction. Note that the tracer distribution of the FDG-PET/CT already incorporates the volume extension caused by breathing displacements from the respective equilibrium, if large respiratory excursions are avoided. If the patient was cooperative, the inaccuracy by quiet breathing can be considered in the range of a few mm. Therefore, a safety margin of 5 mm was considered sufficient, if an appropriate IGRT based on MV-CT was employed.

We checked the intrafractional error by comparing the MV-CT prior to the radiotherapy fraction with an additional MV-CT after irradiation. In our random examinations we found acceptably low deviations of a few mm, which are well considered by the specified safety margins (including the volume enlargement inherent in the PET dataset). We are aware that gating techniques might further reduce the error caused by respiratory motion. However, in case of helical Tomotherapy a gating technique is not available and probably by hardware reasons not possible. Moreover the benefit of gating might be marginal for target volumes of dimensions >5 cm and the specified safety margins.

### 3.3. Outcome

**Figure 2:**
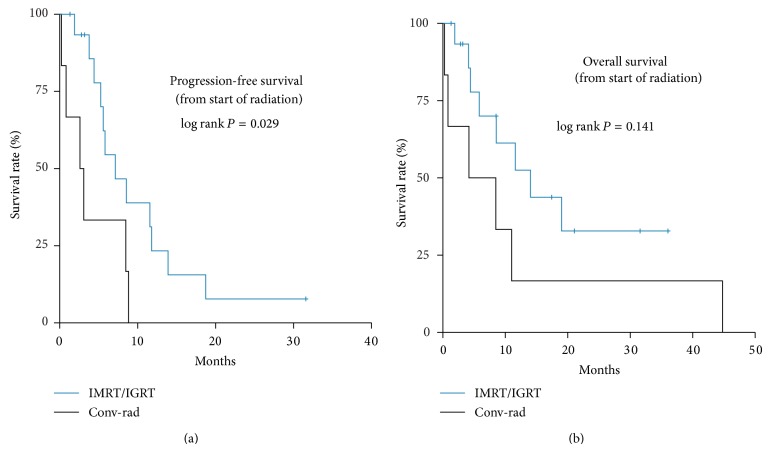
PFS and OS IMRT/IGRT versus conventional radiotherapy calculated from the start of RT.

Median RT-specific progression-free survival was estimated from the beginning of radiation therapy at 5.8 months (95% CI, 3.2–8.4), 2.6 months (95% CI, 0–5.3) in the conventional RT group, and 7.1 months (95% CI, 3.7–10.8) in the IMRT/IGRT group (*P* = 0.029). Median RT-specific overall survival estimated from the beginning of RT was 11.0 months (95% CI, 7.0–15.0), 4.2 months (95% CI, 0–13.3) conventional RT, and 14.0 months (95% CI, 5.2–22.8) IMRT/IGRT (*P* = 0.141); see Figure 2.

**Figure 3:**
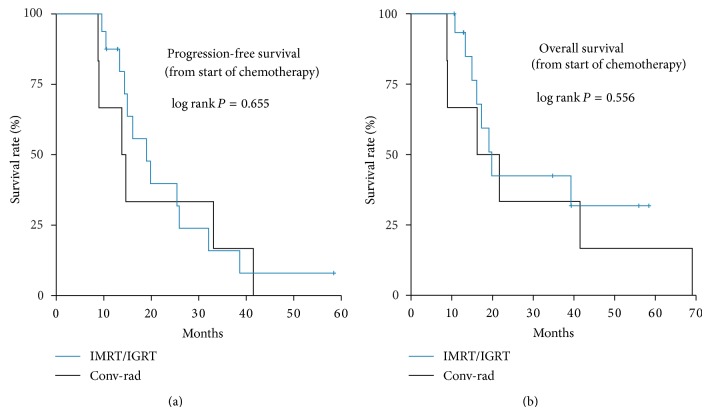
PFS and OS IMRT/IGRT versus conventional radiotherapy calculated from the start of palliative chemotherapy.

If median PFS was based on the start of palliative chemotherapy as general PFS, 16.1 months were reached (95% CI, 10.1–22.1), 13.8 months (95% CI, 7.0–20.6) conventional RT, and 19.0 months (95% CI, 12.6–25.3) IMRT/IGRT (*P* = 0.655). Median general OS was estimated to be 19.8 months (95% CI, 13.9–25.7), 16.2 months (95% CI, 0.9–31.5) conventional RT, and 19.8 months (95% CI, 15.7–23.9) IMRT/IGRT (*P* = 0.556); see Figure 3.

6 patients (27%) had stable disease, 11 patients (50%) had documented distant progressive disease, and 3 patients (14%) had clinical progressive disease. No local progression occurred. 2 patients were lost to follow up. At the time of data bank closure, 8 patients (36%) were still alive and 14 patients (64%) had died.

For the 17 patients who underwent the complete course of RT (85%), median RT-specific PFS was 8.5 months (95% CI, 3.6–13.5), and median RT-specific OS was 14.0 months (95% CI, 2.4–25.6). The median general PFS and OS estimated from the start of palliative first-line chemotherapy were 19.8 months (95% CI, 13.2–26.4) and 21.7 months (95% CI, 0–43.6) respectively.

**Figure 4:**
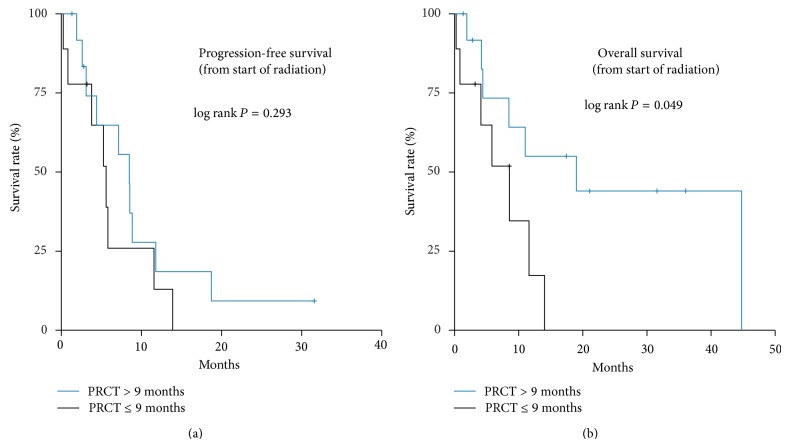
PFS and OS calculated from the he start of RT: duration of preradiation chemotherapy > 9 months versus ≤ 9 months.

With regard to the duration of preradiation chemotherapy, median RT-specific PFS was 8.5 months (95% CI, 6.2–10.7) for patients with PRCT > 9 months compared to 5.6 months (95% CI, 3.2–8.0) for patients with PRCT duration of ≤ 9 months (*P* = 0.293). This effect was translated into a significantly better median RT-specific overall survival for the patients in the PRCT >9 months group, with 19.0 months (0–41.0) compared to 8.5 months (95% CI, 3.3–13.8) in the PRCT < 9 months group (*P* = 0.049); see Figure 4.

## 4. Discussion

22 patients treated between 2005 and 2013 were included in our retrospective analysis investigating the feasibility and efficacy of chemotherapy and subsequent radiation therapy in locally advanced pancreatic cancer. Patients were considered eligible for the evaluation if they had unresectable pancreatic cancer (primary or locally recurrent) without any evidence of distant metastases. This small sample size is disappointing at first glance for a high-volume pancreatic cancer center and has to be seen as the result of the lack of evidence on the role of radiotherapy in LAPC. This fact must be considered a limitation of the above analysis. Our analysis investigated the role of PRCT, especially in regard to optimal duration and comparative effectiveness and toxicities for conventional as opposed to image-guided and intensity-modulated radiation. Median duration of PRCT in our study group was 11 months, considerably higher than in completed or currently recruiting studies for LAPC patients [6]. We were able to demonstrate that effectiveness, toxicity, and treatment adherence were better in the IMRT/IGRT group: two-thirds of the patients in the conventional RT group had to stop RT prematurely, whereas in the IMRT/IGRT group all but one patient were able to complete the planned treatment schedule. This fact translated into a significantly better radiotherapy-related progression-free survival and a lower toxicity profile. Patients who tolerated the planned dosage had a median progression-free survival of 19.8 months and a median overall survival of 21.7 months, which is almost twice as long what could be expected with chemotherapy alone or chemoradiation without induction chemotherapy [2].

Furthermore, our data show that prolonged preradiation chemotherapy (>9 months) translated into a significantly better radiotherapy-specific overall survival (estimated from the start of RT).

Contradictory results for the benefit of RT after induction chemotherapy (ICT) are available, and no clear standard for LAPC has so far been established. On review of the literature, treatment with RT alone, chemoradiation alone or in combination with induction chemotherapy has all been reported. The use of induction chemotherapy was analyzed retrospectively in 181 patients from the GERCOR studies, and a benefit in overall survival for LAPC patients with disease stabilization after an initial chemotherapy period and subsequent radiochemotherapy suggested [6]. In a prospective phase II study, induction chemotherapy consisting of the EGFR-directed antibody cetuximab and the cytotoxic drugs gemcitabine and oxaliplatin was investigated. 69 patients were included in the study. 60 patients received chemoradiation (50.4 Gray in combination with capecitabine 825 mg/m² bid) after the initial chemotherapy, and the median overall survival of 19.2 months was encouraging [9]. Additionally, smad4, a tumor suppressor gene, was evaluated by immunostaining and correlated with prognosis and the pattern of metastatic spread. Kim et al. were able to show similar results in a phase II study investigating ICT with gemcitabine and cisplatin in 37 patients, 25 of whom qualified for RCT, resulting in a median overall survival of 16.8 months [10]. Ch'Ang et al. investigated ICT with gemcitabine, oxaliplatin, and leucovorin/5-FU in 30 eligible patients. Median PFS and OS estimated from the start of ICT were encouraging as well, at 9.3 and 14.5 months, respectively [11].

The implication of these results that induction chemotherapy could be beneficial have recently been weakened by the results of the LAP07 study, which was presented as late-breaking abstract at the 2013 Annual ASCO meeting [12]. This randomized phase III trial could not confirm the expected benefit of induction chemotherapy with gemcitabine +/− erlotinib for 3 months followed by RT (50.4 Gray in combination with capecitabine 800 mg/m²/d), compared to chemotherapy alone with gemcitabine +/− erlotinib. The median OS was similar in both groups, at 16.5 months in the chemotherapy group and 15.3 months in the induction chemotherapy + RCT group, and even slightly higher in the CT group. Full publication must be awaited for a definitive judgment of the data. The current German S3-guideline [13], with the recommendation that “LAPC can be treated by chemotherapy or RCT” reflects the ongoing lack of evidence.

In conclusion, clarification of the role of radiation therapy is still necessary, although the concept of induction chemotherapy for selection of patients without rapid distant tumor spread still appears to be the most convincing strategy.

*Our Data May Help to Answer the Main Remaining Questions.* What are the preferable doses, dose distributions, and methods for a course of RT? How long should chemotherapy be administered, and is there a need for a prolonged chemotherapy phase? What kind of induction chemotherapy is the most promising? According to an American-French Consensus, either three-dimensional conformal radiation or intensity-modulated image-guided radiotherapy with a total dose from 50 to 54 Gray [4] is recommended. Although the significance of our data is limited due to the small sample size, our analysis serves to underline the need for a more precise RT, such as one, ideally, based on IGRT and the attempt to attain higher doses than 50 Gray in the macroscopic parts of the involved region. This can be realized by simultaneous integrated boost (SIB) to the gross tumor volume. This concept needs to be defined more precisely by future prospective trials. 

Regarding the type of induction chemotherapy, the investigator-initiated trial CONKO-007 [14] will help to clarify whether a more intense induction of 12 weeks chemotherapy with the use of FOLFIRINOX, which so far provides the best response rates for metastatic pancreatic cancer [15], has a clear effect in patients with LAPC and subsequent RT. Our data suggest that radiotherapy of more than 3 months duration may be more effective, but this concept also needs to be analyzed in future trials.

In contrast to earlier convictions (subsuming metastatic spread as mean cause of death in pancreatic cancer), the landmark study of Yachida et al. [16] suggests that about a third of patients died due to local persistence of the disease; so local disease control must be considered an equally relevant factor for the prognosis of pancreatic cancer patients. In future it may be possible to identify those patients with LAPC likely to receive maximum benefit from RT and those eligible for more aggressive local treatments by using biomarkers such as smad4 to stratify patients into “rapid metastasizers” and “slow metastasizers” [17]. Identifying additional prognostic factors would be of major interest for further clinical investigations.

This possible use of prognostic biomarkers as potential predictors of distant metastasis in pancreatic cancer to select patients for prolonged and more effective induction chemotherapy protocols and for more sophisticated application of irradiation therapy of sufficiently high doses gives hope for more detailed therapeutic concepts and for the future. This therapeutic concept may help to improve the still dismal prognosis in selected patients with LAPC.

## 5. Conclusion 

Patients with locally advanced inoperable pancreatic cancer who did not metastasize under prolonged first-line chemotherapy may profit from subsequent radiochemotherapy after disease stabilization. The combination of preradiation chemotherapy for longer than 9 months and radiotherapy using modern techniques such as intensity-modulated radiotherapy and image-guided radiotherapy provides encouraging therapeutic results. Further studies are needed to prove this concept in patients with LAPC.

## Figures and Tables

**Table 1:** Patient's characteristics and treatment details.

	All patients	Conv-rad	IMRT/IGRT
	*N* = (%)	*N* = (%)	N = (%)
Age			
Median	64 (40–77)	65 (53–77)	62 (40–74)
♀/♂	15/7 (68/32)	5/1 (83/17)	10/6 (62.5/37.5)
Previous resection			
Yes/no	10/12 (45/55)	4/2 (67/33)	8/8 (50/50)
Tumor localisation			
Pancreatic head	16 (73)	4 (67)	11 (69)
Body	5 (22)	1 (17)	4 (25)
Tail	1 (5)	1 (17)	1 (6)
Radiation therapy			
Alone	10 (45)	4 (67)	6 (37.5)
With chemotherapy	12 (55)	2 (33)	10 (62.5)
3D conformal	6 (27)	6 (100)	—
IMRT/IGRT	16 (73)	—	16 (100)
Preradiation chem			
GemMono	20 (91)	5 (83)	15 (94)
GemCombination	2 (9)	1 (17)	1 (6)
>1 therapy	5 (22)	3 (50)	2 (13)
Duration preradiation chemotherapy			
3–6 months	3 (14)	0	3 (19)
6–9 months	6 (27)	2 (33)	4 (25)
9–12 months	3 (14)	1 (17)	2 (12)
>12 months	10 (45)	3 (50)	7 (44)

## Abbreviations

Conv-RT: Conventional radiotherapy
CT: Chemotherapy
CTV: Clinical target volume
GTV: Gross tumor volume
Gy: Gray
ICT: Induction chemotherapy
IGRT: Image-guided radiotherapy
IMRT: Intensity-modulated radiotherapy
LAPC: Locally advanced pancreatic cancer
MV-CT: Megavoltage computed tomography
OS: Overall survival
PFS: Progression-free survival
PRCT: Preradiation chemotherapy
Pts: Patients
PTV: Planning target volume
RT: Radiotherapy
RCT: Radiochemotherapy
RT: Radiation therapy
SIB: Simultaneous integrated boost.
